# Sex differences in early transcriptomic responses to oxidative stress in the copepod *Tigriopus californicus*

**DOI:** 10.1186/s12864-020-07179-5

**Published:** 2020-11-03

**Authors:** Ning Li, Ben A. Flanagan, MacKenzie Partridge, Elaine J. Huang, Suzanne Edmands

**Affiliations:** Department of Biological Sciences, University of Southern California, 3616 Trousdale Parkway, Los Angeles, CA 90089 USA

**Keywords:** RNA-seq, Hydrogen peroxide, Paraquat, Tolerance, Antioxidant

## Abstract

**Background:**

Patterns of gene expression can be dramatically different between males and females of the same species, in part due to genes on sex chromosomes. Here we test for sex differences in early transcriptomic response to oxidative stress in a species which lacks heteromorphic sex chromosomes, the copepod *Tigriopus californicus*.

**Results:**

Male and female individuals were separately exposed to control conditions and pro-oxidant conditions (hydrogen peroxide and paraquat) for periods of 3 hours and 6 hours. Variance partitioning showed the greatest expression variance among individuals, highlighting the important information that can be obscured by the common practice of pooling individuals. Gene expression variance between sexes was greater than that among treatments, showing the profound effect of sex even when males and females share the same genome. Males exhibited a larger response to both pro-oxidants, differentially expressing more than four times as many genes, including up-regulation of more antioxidant genes, heat shock proteins and protease genes. While females differentially expressed fewer genes, the magnitudes of fold change were generally greater, indicating a more targeted response. Although females shared a smaller fraction of differentially expressed genes between stressors and time points, expression patterns of antioxidant and protease genes were more similar between stressors and more GO terms were shared between time points.

**Conclusions:**

Early transcriptomic responses to the pro-oxidants H_2_O_2_ and paraquat in copepods revealed substantial variation among individuals and between sexes. The finding of such profound sex differences in oxidative stress response, even in the absence of sex chromosomes, highlights the importance of studying both sexes and the potential for developing sex-specific strategies to promote optimal health and aging in humans.

**Supplementary Information:**

The online version contains supplementary material available at 10.1186/s12864-020-07179-5.

## Background

Oxygen, as a necessity to aerobic organisms, acts as a terminal site for accepting electrons in respiration, and at the same time, works as a generator of dangerous side products – reactive oxygen species or ROS [1]. Aerobic organisms are frequently exposed to various endogenous and environmental conditions which give rise to the accumulation of ROS. Aquatic organisms are faced with a particularly high number of ROS sources, including variation in temperature, salinity and oxygen level, as well as other factors such as heavy metal ions, pesticides, herbicides and oil-related pollutants [2]. Accumulated ROS may damage proteins, lipids, DNA, RNA and other cellular components, thereby resulting in severe physiological dysfunction [3, 4]. Consequently, organisms have developed a series of ROS scavenging systems, including antioxidant enzymes such as superoxide dismutase and catalase and non-enzymatic antioxidants such as glutathione and ascorbic acid [1, 2].

Usually ROS levels are maintained at a dynamic and steady state, with the continuous generation of ROS being balanced by continuous elimination [2]. This steady state concentration is favored since some kinds of ROS (e.g. H_2_O_2_) have been found to play important roles in regulation of cellular processes as signaling molecules and also protect against infection [5–7]. However, when ROS levels are enhanced, a disturbance of redox status, termed oxidative stress, will be triggered. The antioxidant system will then be provoked to scavenge the excessive ROS to prevent oxidative stress-induced cellular damage, and eventually the ROS level will decrease to a steady-state level, during which the expression of hundreds or thousands of genes may be affected [7]. Oxidative stress not only induces ROS detoxification-related gene expression, such as genes encoding antioxidant enzymes, but also influences the expression of genes involved in signal transduction, protein or lipid metabolism and transcriptional or translational regulation (e.g. [8–11]).

The effects of oxidative stress have been proven to differ substantially between sexes. Under exposure to ethanol, *Drosophila melanogaster* females exhibited higher resistance to oxidative stress as evident from lower mortality and ROS levels as well as higher activities of antioxidant enzymes [12]. Similarly, studies in rats have found higher protection from antioxidant enzymes and lower oxidative damage in females [13–15]. Furthermore, as reviewed in multiple studies, the sex-specific effects of oxidative stress might play a critical role in sex differences in development of various diseases and lifespan [16, 17]. Sex-biased responses to oxidative stress may be particularly relevant to sex-specific aging, due to the relationship between oxidative stress and aging and the correlation between oxidative stress tolerance and long lifespan [12, 18–21]. Thus, measuring transcriptional responses to oxidative stress in both sexes will not only help elucidate sex-specific oxidative stress response systems, but also shed light on the molecular mechanisms of sex differences in longevity.

This study is focused on the intertidal copepod *Tigriopus californicus*, an ideal lab model due to its short generation time, easy culturing and extensive genetic and genomic resources [22]. The species is also an alternative model for sex-specific studies due to the absence of sex chromosomes [22–26], where sex differences will not be complicated by dosage compensation or sexual conflict [27–31]. Instead, sex determination is polygenic with a small environmental component [23–26]. Despite the absence of sex chromosomes in this species, substantial sex differences have been documented, with males showing lower tolerance to a wide range of stressors [32–34]. Our previous study successfully applied single-individual RNA-seq to this species for the first time, in an analysis of sex-specific transcriptomic responses to long-term oxidative stress induced by 35 days of exposure to hydrogen peroxide (H_2_O_2_) and a yeast diet [35]. Our results showed that less tolerant male copepods differentially expressed more genes in response to the long-term oxidative stress treatments while females differentially expressed fewer genes but with larger magnitudes of fold change. Here we test for sex differences in response to short-term oxidative stress. While this study did not directly measure oxidative stress (such as by ROS levels), it used two chemicals, hydrogen peroxide and paraquat that are well-established as pro-oxidants. Because transcriptomic response to oxidative stress in other species has shown dramatic temporal changes [11, 36], sex-specific responses in *T. californicus* in the first few hours of exposure may be very different from those after a month of exposure.

Sex-specific response may also differ substantially between exposures to different pro-oxidants. In *Drosophila melanogaster*, Pomatto et al. [37] found that females but not males acclimated to H_2_O_2_ stress, whereas males but not females acclimated to paraquat (N,N′-dimethyl-4,4′-bipyridinium dichloride) stress. H_2_O_2_ is a commonly used stressor for oxidative stress exposure since it is produced by the mitochondrial electron-transport chain and directly increases the ROS level [1, 38]. Paraquat is an herbicide, and its toxicity comes from the generation of superoxide anion which can form more ROS such as hydrogen peroxide and hydroxyl radical [39, 40]. Given previous evidence for contrasting sex-specific responses to H_2_O_2_ and paraquat, we assessed sex differences in transcriptomic responses to these same chemicals. While most transcriptomic studies in *T. californicus* have pooled hundreds of individuals to increase the total amount of RNA [41–46], this can greatly increase pooling bias and false discovery rate [47–49]. Here we instead used single-individual RNA-seq to assess individual variation in stress response, which was shown to be substantial in our previous work [35]. To assess temporal dynamics of an initial stress response, gene expression was characterized at two different time points (3 h and 6 h).

## Results

### Single-individual RNA-seq

To assess sex-specific transcriptomic responses to oxidative stress, we performed single-individual RNA-seq on both sexes after exposure to two stressors (H_2_O_2_ and paraquat) for 3 h and 6 h. Four biological replicates were used for all treatments at each time point within each sex. A total of 48 individual samples were sequenced on two lanes, yielding ~ 917.7 million 150 bp read pairs. After trimming adapters, low quality bases (base quality < 20) and short reads (trimmed length < 36 bp), ~ 824.8 million read pairs were retained ranging from 9.9 to 27.4 for each individual (Table S1). Clean reads were mapped to annotated genes in the reference genome, resulting in 39.5% mapping efficiency on average (Table S1). The read sequences were deposited at the National Center for Biotechnology Information (NCBI) Sequence Read Archive (SRA) under the accession numbers SRR10444974 – SRR10445021.

Alignment of the non-mapped reads revealed contamination from bacteria, fungi, algae and amplified genomic DNA from other species studied in the lab. This contamination is likely aggravated by the library preparation protocol, which requires twenty cycles of PCR. Despite the contamination, the quality of the mapped sequence data is high. The average number of mapped read pairs per sample is just under 7 million (Table S1), which is higher than our previous transcriptome study and well above the sequencing depth recommended for human RNA-seq studies, after adjusting for genome size (ENCODE Guidelines and Best Practices for RNA-Seq: Revised December 2016). Our power to identify DEGs is further supported by the use of four replicates per treatment. Together, the sequencing depth and replication in this study are more than sufficient for analyses of the moderately or highly expressed protein coding genes [50, 51]. In addition, we filtered out genes with less than or equal to 50 total counts across all the individuals and 13,739 genes (87.8% of total genes) remained, indicating the relatively satisfying coverage of the genome coding regions.

**Fig. 1:**
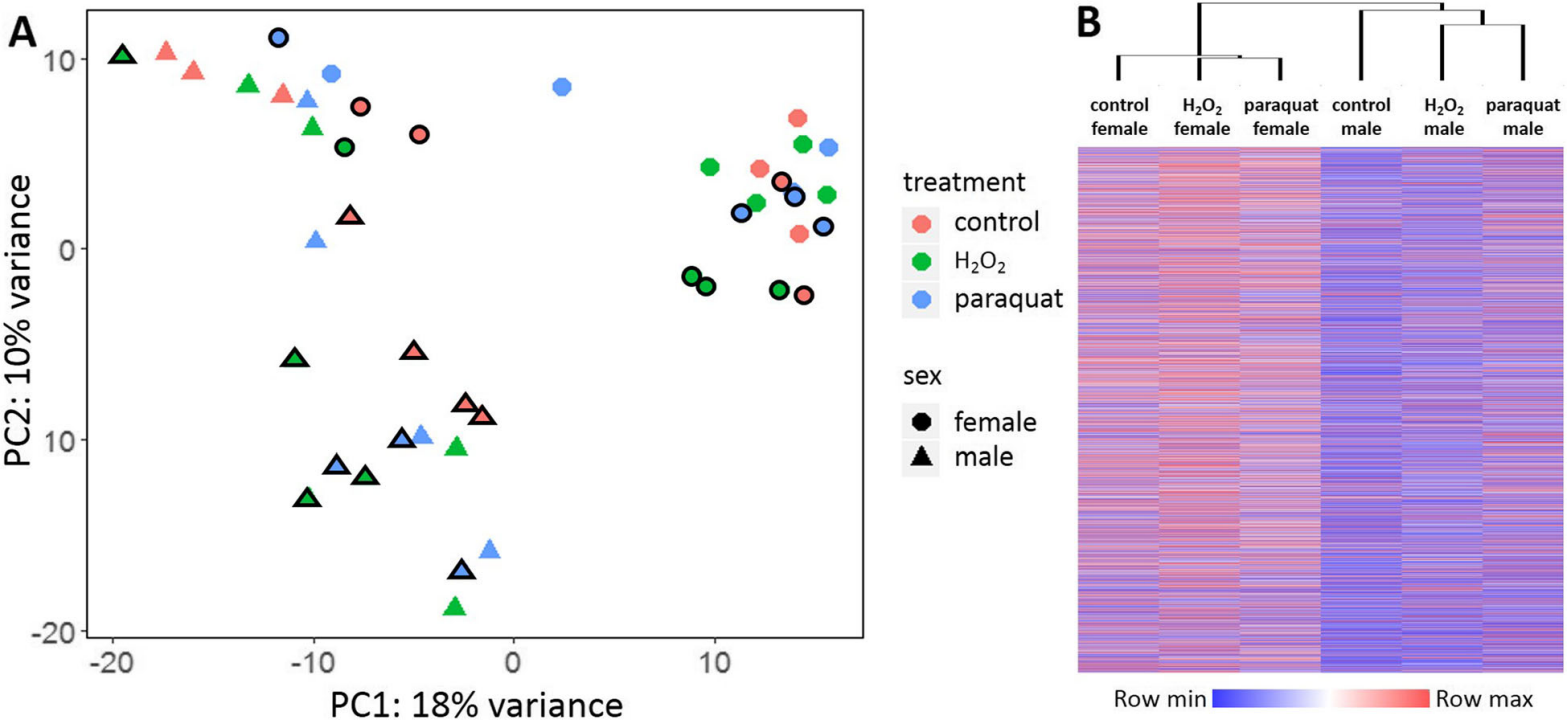
Expression patterns across sexes, treatments and time points. **a** Principal component analysis (PCA) of the top 500 genes in terms of variance across individuals. Information on sex and treatment is indicated in the plot. Samples with a black border were collected at the 3 h time point, and samples without a black border were collected at the 6 h time point. The proportion of variance explained by the first two principal components is indicated beside the two axes. **b** Heatmap of average transformed expression values across all groups. The two time points were combined for each group to show the effects of sex and treatment because they did not show overall apparent differences in gene expression. Expression values were transformed by the vst function in DESeq2. Hierarchical clustering was conducted using one minus Pearson correlation in Morpheus. For each gene, high relative expression is indicated by warmer colors (red), and low relative expression is indicated by colder colors (blue)

Principal component analysis (PCA) was conducted to show relationships among the 48 samples and identify possible outliers. One female and one male under the control condition at 6 h, and one male under the paraquat condition at 3 h were removed as outliers from further analyses. The resultant PCA (Fig. 1a) showed that 18.0% of the variance among samples is attributed to PC1, and 10.0% to PC2. Males and females were separated by both PC1 and PC2 (meaning that a diagonal line can be drawn to separate the two sexes), regardless of treatments and time points, except for one female under the H_2_O_2_ treatment at 3 h. No obvious effect of treatment or time point was observed in the PCA plot. Sample clustering analysis (Fig. 1b) also showed that sex differences were greater than treatment differences with the two sexes forming two separate clusters. Additionally, the two treatment groups were clustered together within each sex, while separate from control groups (Fig. 1b). ANOVA revealed that, on average, at 3 h 37.0, 54.0 and 9.0% of the gene expression variation, and at 6 h 51.1, 42.9 and 6.0% of the gene expression variation were explained by variations between sexes, among individuals and among treatments, respectively.

### Differential expression analysis between sexes within treatments

**Fig. 2:**
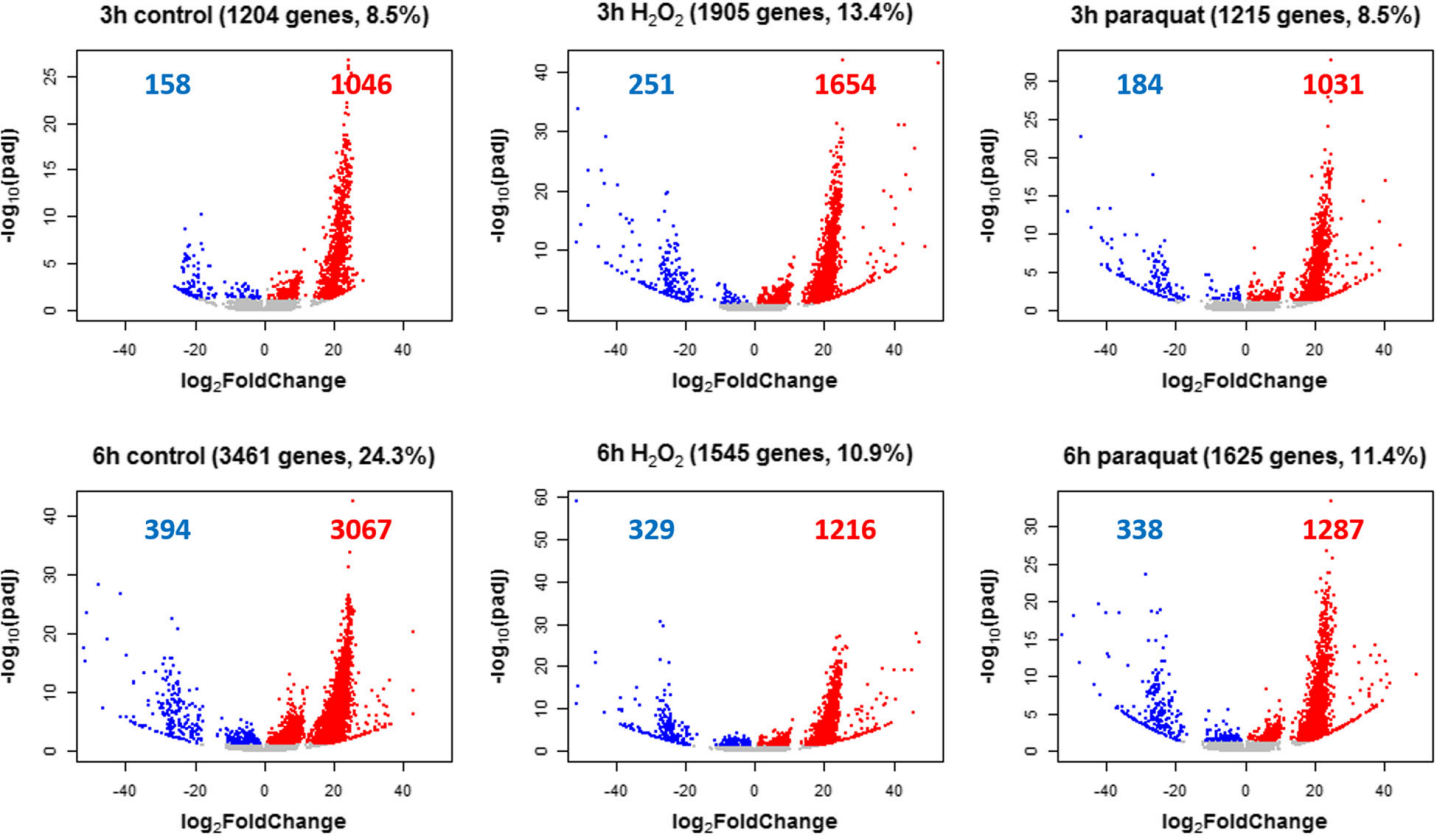
Volcano plots showing the sex-biased gene expression within each group. The number and percentage (relative to all the genes analyzed in this study) of sex-biased genes are indicated in the titles of each plot. Red dots represent highly expressed genes in females (female-biased gene expression) and blue dots represent highly expressed genes in males (male-biased gene expression). The number of both female and male biased genes is indicated with the same color in the plots

The two sexes were compared under each treatment at each time point to assess sex-bias in gene expression. Of the 14,230 genes analyzed here, 8.5–24.3% of the genes showed sex-biased expression within treatments (Fig. 2). Within each treatment, 78.7–88.6% of the genes with sex-biased expression are female-biased (For the same gene, females have higher expression levels than males; Fig. 2). Under oxidative stress, less than 37.0% of the sex-biased genes were shared by both treatments (445 at 3 h, 421 at 6 h), indicating that sex-biased gene expression is stressor-specific.

### Differential expression analysis between treatments within sexes

**Fig. 3:**
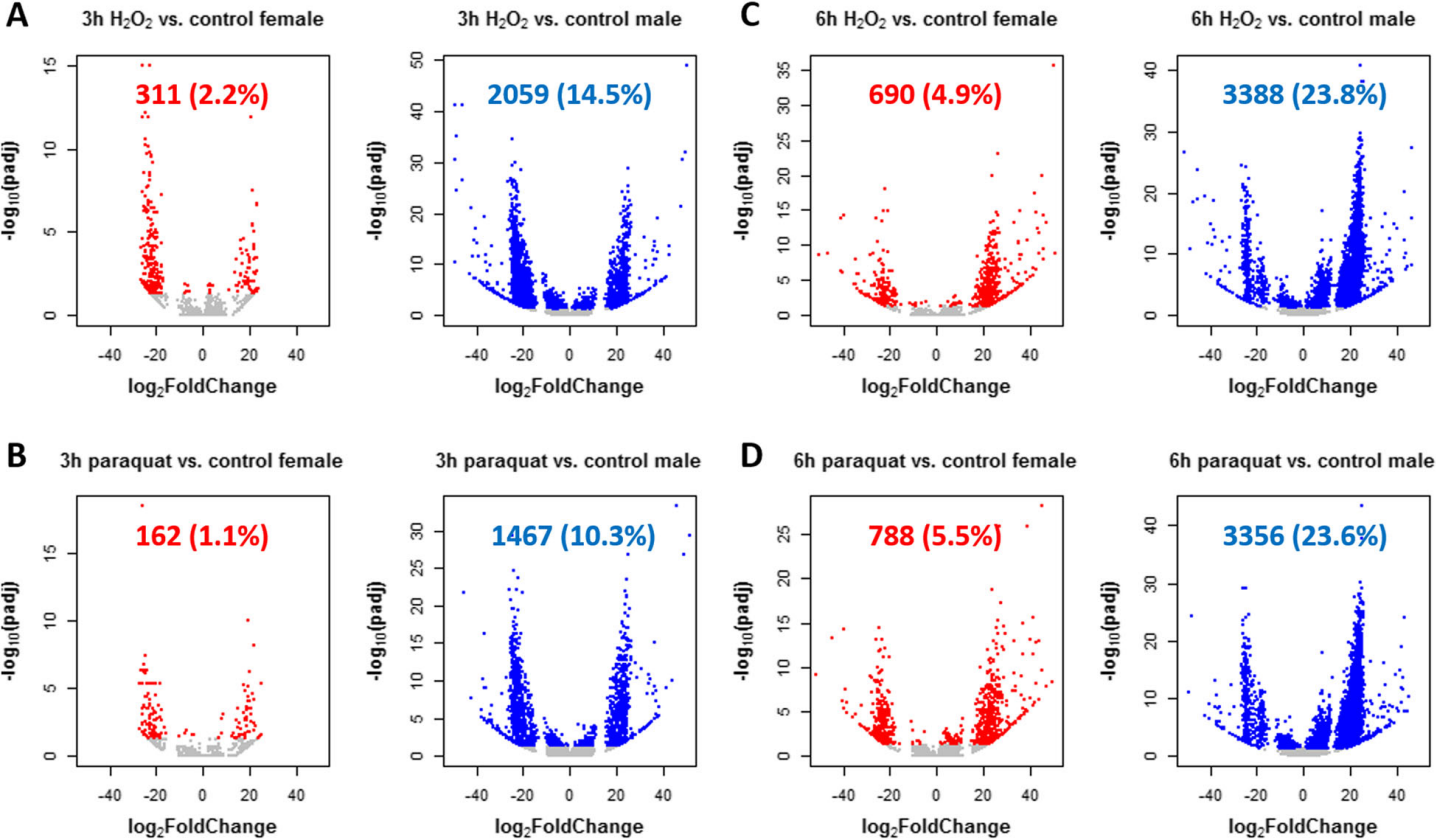
Volcano plots showing differentially expressed genes (DEGs) within each sex under each treatment at each time point. **a** DEGs under H_2_O_2_ treatment at 3 h. **b** DEGs under paraquat treatment at 3 h. **c** DEGs under H_2_O_2_ treatment at 6 h. **d** DEGs under paraquat treatment at 6 h. The number and percentage (relative to all the genes analyzed in this study) of DEGs are indicated in the plots. Red dots represent DEGs in females and blue dots represent DEGs in males

**Fig. 4:**
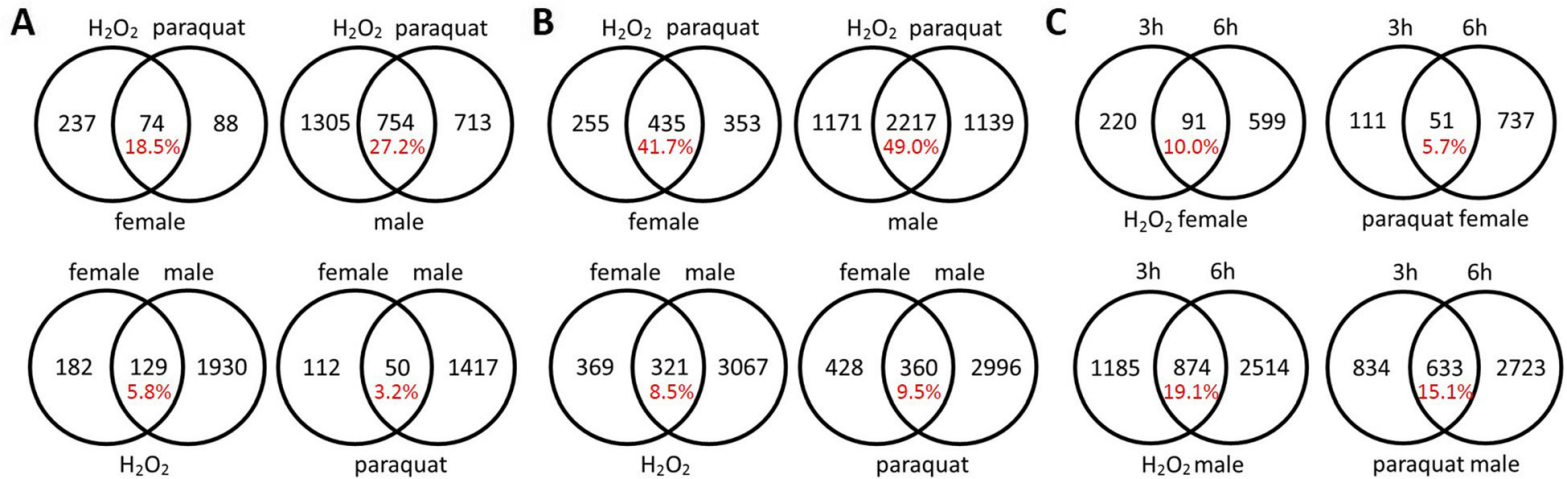
Venn diagrams showing the number of differentially expressed genes shared between contrasts at 3 h (**a**), at 6 h (**b**) and between the two time points (**c**)

The analyses focused on comparisons to the control at the same time point to avoid complications due to batch effects of sequencing. To understand sex differences in gene expression responding to short-term oxidative stress, comparisons between treated groups and control groups were performed within each sex at each time point. Sex differences were observed with males differentially expressing more genes (between 81.0 and 91.0% of the total DEGs) than females under each stress treatment at each time point (Fig. 3). Analysis of the percentage of shared DEGs between contrasts at each time point (Fig. 4) showed that the two treatment groups shared a greater proportion of DEGs within sex than that shared by two sexes within treatment. Both findings indicated that the sex difference is a major source of variation in gene expression, consistent with the results from the PCA and the sample clustering analysis. At each time point, the fold changes of genes differentially expressed under either H_2_O_2_ or paraquat within each sex were collected for comparisons. At the 3 h time point, the females had average absolute fold change values of 16.9 and 10.8 while the males had means of 14.5 and 11.8 under H_2_O_2_ (Mann-Whitney-Wilcoxon test between sexes, *p* = 4.99e – 05) and paraquat treatments (*p* = 0.21), respectively. Also, at the 6 h time point, the females displayed mean values of 17.1 and 18.2 while the males averaged 13.5 and 13.2 under H_2_O_2_ (*p* = 2.2e – 16) and paraquat treatments (*p* = 2.2e – 16), respectively. Thus, under short-term oxidative stress, females differentially expressed fewer genes in all four comparisons but with greater magnitudes of fold change in three out of four comparisons.

Further, more DEGs were identified at 6 h than those found at 3 h within each sex under each treatment (Fig. 3). In terms of percentages of up-regulation and down-regulation, individuals at 6 h up-regulated more genes than those at 3 h under the same treatment within the same sex (Table 1). Within time points (Fig. 4a and b), females shared a smaller proportion of DEGs between stress treatments than males did, indicating a more stressor-specific response. Between time points (Fig. 4c), females also shared a smaller proportion of DEGs than males did, indicating a more temporally dynamic response.

**Table 1:** The number and percentage of differentially expressed genes within each comparison

	3 h time point			6 h time point		
	Up	Down	Total	Up	Down	Total
H_2_O_2_ vs. control in females	72	239	311	496	194	690
	(23.2%)	(76.8%)		(71.9%)	(28.1%)	
Paraquat vs. control in females	57	105	162	519	269	788
	(35.2%)	(64.8%)		(65.9%)	(34.1%)	
H_2_O_2_ vs. control in males	746	1313	2059	2836	552	3388
	(36.2%)	(63.8%)		(83.7%)	(16.3%)	
Paraquat vs. control in males	701	766	1467	2724	632	3356
	(47.8%)	(52.2%)		(81.2%)	(18.8%)	

### GO enrichment analysis

Up-regulated and down-regulated genes within each contrast were used for GO enrichment analyses separately (Tables S2, S3, S4, S5). The GO enrichment results were further investigated to show enriched GO terms shared between groups (Table S6). Both sexes up-regulated peptide catabolic process (GO:0043171) and peptidase activity (GO:0004175) at 3 h. At 6 h, peptidase activity (GO:0070011) was still up-regulated in males but down-regulated in females. Hydrolase activity (GO:0016787) was found to be up-regulated in females at 3 h and in males at 6 h. Also, both sexes up-regulated synaptic signaling (GO:0098916, GO:0099536, GO:0099537) only at 6 h. In females, pyruvate and lactate transport related GO terms (GO:0006848, GO:1901475, GO:0015727, GO:0035873, GO:0015129) were up-regulated, while renal function (GO:0072127, GO:0072128, GO:0072129, GO:0072130), gonadotropin secretion (GO:0032274) and luteinizing hormone secretion (GO:0032275) were down-regulated at both time points. However, no GO terms were shared by both time points in males, while at the later time point, more oxidative stress responding GO terms, such as autophagy (GO:0010508), protein repair (GO:0030091) and catalytic activity (GO:0003824) were up-regulated.

### Identification of oxidative stress responding genes

Oxidative stress has been well studied, and a wide range of genes have been identified to respond to or scavenge ROS. Based on the available gene annotation information for *T. californicus* [22], we searched our DEG lists for antioxidant genes identified in previous work [52–54]. We searched in particular for heat shock proteins, which were known to respond to oxidative stress in *Tigriopus* (e.g. [55]) and for protease genes, since *T. californicus* has been shown to exhibit sex differences in proteolytic activity following stress exposure [34]. Table 2 listed 12 gene categories found to be differentially regulated. Notably, catalase, glutathione reductase and thioredoxin reductase were not found to be differentially expressed in any group.

**Table 2:** The number of differentially expressed oxidative stress responding genes

Gene category		3 h time point				6 h time point			
		H_2_O_2_ female	Paraquat female	H_2_O_2_ male	Paraquat male	H_2_O_2_ female	Paraquat female	H_2_O_2_ male	Paraquat male
Antioxidant	Superoxide dismutase	- / -	- / -	- / -	- / -	- / -	- / -	2 / -	2 / -
	Glutathione peroxidase	- / -	- / -	- / -	- / -	- / -	- / -	1 / -	1 / -
	Glutathione S-transferase	- / -	- / -	2 / 3	4 / -	3 / -	3 / -	6 / -	4 / 1
	Glutathione dehydrogenase	- / -	- / -	- / -	- / -	- / -	- / -	1 / -	1 / -
	Thioredoxin	- / -	- / -	- / -	- / -	- / -	- / -	2 / -	2 / -
	Thioredoxin domain containing protein	- / -	- / -	- / -	- / 1	- / -	- / -	1 / -	- / 1
	Peroxiredoxin	- / -	- / -	- / -	- / -	- / -	- / -	3 / -	2 / -
	Oxidoreductase	- / 1	- / -	2 / 1	1 / -	- / -	1 / -	3 / 2	2 / 2
	Mitogen activated protein kinases	- / -	- / -	1 / -	1 / 1	- / -	- / 2	5 / -	7 / -
	Ascorbate peroxidase	- / -	- / -	1 / -	1 / -	1 / -	1 / -	- / -	1 / -
Heat shock protein		- / -	- / 1	1 / 1	3 / -	- / -	1 / -	5 / 1	6 / -
Protease		- / -	- / -	- / 6	- / 3	2 / -	2 / -	9 / 2	13 / 3

Number of differentially expressed genes is displayed as “no. up-regulated genes / no. down-regulated genes”, with “-” indicating that no genes were found

For the antioxidants and heat shock proteins (Table 2), the vast majority of DEGs were up-regulated. More up-regulated genes were found in male groups than female groups and more in 6 h groups than 3 h groups. No gene up-regulation was found in female groups at 3 h. For protease genes (Tables 2 and S7), at the 3 h time point no proteases were differentially expressed in females, while six and three genes (two genes were shared) were down-regulated in males under H_2_O_2_ and paraquat, respectively. However, at the later time point, females up-regulated the same two genes to respond to both stressors, while 11 (2 down-regulated and 9 up-regulated) and 16 (3 down-regulated and 13 up-regulated) genes were differentially expressed in males under H_2_O_2_ and paraquat respectively with 9 genes shared between stressors.

## Discussion

Our previous work [35] used single-individual RNA-seq in the copepod *T. californicus* to assess sex differences in transcriptomic responses after long-term exposure (35 days) to oxidative stressors. The current study took a similar approach to assess sex differences after short-term exposure (3 h and 6 h) to oxidative stressors. Despite the absence of sex chromosomes in this species, both studies found substantial sex differences in gene expression. Both studies showed that sex-biased expression was female-biased within each group, but the proportion of genes with sex biased gene expression (8.5–24.3%) is much lower than the 34.7–42.7% found in another copepod species *Lepeophtheirus salmonis* that possesses sex chromosomes [56]. Since extractions used whole animals instead of specific tissues, sex-biased expression within treatments may be partially driven by differences in the tissue types (such as gonads) and proportions in the two sexes. For both studies, pairwise comparisons between stress treated groups and control groups showed that males differentially expressed more genes than females. These differences may be associated with the strong stress tolerance in females and the weak tolerance in males, reflected by their responses to a wide range of stressors including temperature, salinity and marine pollutants [32–34]. In addition, both studies found that while females have fewer DEGs in response to oxidative stress, the average magnitudes of fold change were generally greater, suggesting a more targeted response in females. These results show that despite the dynamic temporal nature of stress-induced gene expression, consistent sex differences can be found across widely divergent exposure times.

Our finding of a greater number of stress DEGs in the less tolerant type (male copepods) is similar to those found in a number of other systems including corals [57], snails [58] and abalones [59]. Importantly we cannot say whether the lower number of stress DEGs in females, by itself, indicates that females have a more efficient response, or simply experienced a lower level of stress. Our finding of a more targeted response (fewer DEGs but with greater fold change) in the more tolerant type (female copepods) is particularly similar to results of Chen et al. [59], where the more heat-tolerant abalone line differentially expressed fewer genes yet showed greater magnitudes of change for certain key genes including those in the heat shock protein family. Notably, these other studies compared more and less tolerant populations, while our study is the first, to our knowledge, to show such patterns in comparisons between sexes.

Two time points (3 h and 6 h) were used in this study to capture early responses in transcriptomes. The results demonstrated temporal differences with more DEGs and more up-regulation at 6 h than at 3 h. Interestingly, sex differences were also observed in comparisons between the two time points, with females sharing a smaller fraction of DEGs between time points, thereby inducing different gene sets to cope with oxidative stress over time. Moreover, a search of well-known oxidative stress responding genes in each contrast DEG list showed that females at 3 h did not up-regulate any of these known genes, and both males and females up-regulated more of these known genes at 6 h than at 3 h. Results suggested that glutathione S-transferases (GSTs) may be particularly good marker genes for oxidative stress responses since they were highly expressed in both sexes under both stress treatments at 6 h. Notably, GSTs have been identified as important biomarkers for oxidative stress response to H_2_O_2_, heavy metals and high temperature in studies of the congeners *T. japonicus* and *T. kingsejongensis* [60–62], with stress-induced changes in GST levels paralleling changes in ROS levels [60].

While our previous study [35] used one pro-oxidant treatment and one reduced antioxidant treatment, the present study used two pro-oxidants: H_2_O_2_ and paraquat. These chemicals were chosen based on a *Drosophila* study [37], in which females but not males acclimated to H_2_O_2_, while males but not females acclimated to paraquat. Our study instead found that in response to both stressors, males differentially expressed more than four times as many genes as females. Further, the two stressors were found to induce distinct responses within each sex, with only 18.5 and 41.7% overlap in DEGs for females and 27.2 and 49.0% overlap in DEGs for males at 3 h and 6 h respectively. In terms of shared genes between sexes for each stressor, 7.2 and 19.5% overlap were observed at 3 h and 6 h, respectively, suggesting divergence in responding mechanisms induced by H_2_O_2_ versus paraquat.

Sex difference was found to be a major factor affecting gene expression, accounting for 37.0 and 51.1% of the total expression variance at 3 h and 6 h, respectively. Variation among individuals was also substantial, accounting for 54.0 and 42.9% of the total expression variance at the two time points. And finally, differences among treatments were relatively small, making up only 9.0 and 6.0% of the total variance at the two time points. Expression variance among individuals in this study was notably higher than the 27.0% found in our previous study of long-term exposure to oxidative stress [35]. Since no individuals died during the short-term exposure, we hypothesize that the previous long-term exposure eliminated susceptible individuals and thereby reduced individual variation. In both studies, expression variance among individuals was greater than that among treatments highlighting the information that is obscured through the common practice of combining individuals into pooled samples. Since both studies used outbred populations, the observed expression variance may be due to genetic variation within populations. Such variation is important to document, as it is the fuel for natural selection to promote adaptive change in oxidative stress tolerance. Individual variation in gene expression could also be caused by variation in mating and/or reproductive status, by exposure time differences or by individual variation in handling stress, including the removal of eggs sacs from females. While sources of individual variation cannot be partitioned in the current study, the magnitude of individual variance found argues for future work explicitly designed to partition sources of individual variation.

GO enrichment analyses were conducted for DEGs in all the contrasts. Female responses to oxidative stress at 3 h demonstrated up-regulation of hydrolytic activity and peptidolytic activity dealing with damaged cellular components, but these activities seemed to cease at 6 h. Additionally, pyruvate and lactate transport were active across the two time points in females, protecting cells from oxidative stress by activating cellular defense and antioxidant actions [63, 64]. Some effects on neural system were also observed at 6 h in both sexes, suggested by the up-regulation of synaptic signaling. In contrast to females, males continually up-regulated peptidase activity through both time points, and at the later time point activated hydrolase activity, protein repair and autophagy to fix or clean up dysfunctional components, indicating the less efficient responses to oxidative stress in males.

## Conclusion

In summary, early transcriptomic responses to the pro-oxidants H_2_O_2_ and paraquat were profoundly different between males and females. The gene expression variance between sexes was even greater than that among treatments for both time points. In response to oxidative stress, the less tolerant male copepods differentially expressed many more genes, including more up-regulation of antioxidants and heat shock proteins at both time points, and more up-regulation of protease genes at the later time point. While females had far fewer DEGs, their average magnitudes of fold change were greater. Between stress treatments, females shared a smaller proportion of DEGs but more similar expression patterns of antioxidant and protease genes. Between time points, females shared more GO terms but a smaller fraction of DEGs. These overall patterns were consistent with a larger early transcriptomic response in the less tolerant male copepods, with sex differences in the time course of the response and the level of specificity between stressors.

## Methods

### Culture maintenance and generation of age-matched copepods

Copepods were collected from supralittoral splash pools in San Diego, CA (32°74′60″N, 117°25′51″W) during summer 2018. They were confirmed to be *T. californicus* based on habitat (no other copepod species inhabit this extreme environment [65]), morphology and the following transcriptome sequencing data, and were maintained in a 20 °C incubator prior to the start of the experiment. Sea water for culturing was collected from Catalina Island and salinity ranged from 32 to 34 parts per thousand (ppt). Generation of age-matched animals was facilitated by the well-known reproductive biology of this species [66], with year-round reproduction and no evidence of seasonality. Male *T. californicus* clasp immature females (copepodid stages II-V) prior to the female’s terminal molt, and the female is inseminated and released upon maturation (stage VI) [66]. Females only mate once during their lifetime and produce successive fertilized egg sacs from stored sperm [66]. To generate cohorts of copepods with known age, 90 gravid and healthy females with orange egg sacs were isolated and held at 20 °C overnight to allow eggs to hatch. Females with orange eggs were targeted because the orange coloration is indicative of late-stage development prior to hatching. Upon hatching, larvae were incubated at 20 °C on a 12 h:12 h light:dark cycle and grown into adults after six naupliar stages and five copepodid stages. They were allowed to develop for 4 weeks or until gravid females were observed and then male and female adults (distinguishable by their antennule structures) were separated into two dishes. Notably, a subset of animals is expected to have mated prior to separation of two sexes. All adult animals were transferred to a new dish weekly to remove newly hatched larvae and thereby prevent mixing generations. Throughout, animals were fed weekly using a mixture of powdered Spirulina (Nutrex Hawaii, USA) and ground Tetramin flakes (Tetra, Germany) at a concentration of 0.1 g of each food per 1 L filtered seawater (37 μm).

### Oxidative stress treatment and sample collection

Three media were prepared for oxidative stress treatments: (1) 1.2 mM H_2_O_2_ treatment: 12.3 μL 30.0% H_2_O_2_ solution (EMD Millipore, Germany) was added to 100 mL filtered seawater; (2) 80 ppm (parts per million, 1 ppm = 0.0001%) paraquat treatment: 0.1 g paraquat (Sigma-Aldrich, USA) was added to 1 mL deionized water to make 100,000 ppm solution and then 80 μL was transferred to 100 mL filtered seawater; and (3) control condition: filtered seawater only. The concentrations of H_2_O_2_ and paraquat were determined by averaging the mean 24 h LC50 values (the lethal concentration required to kill 50.0% of the population) for both males and females from the San Diego population (unpublished data). Treatments used eight-week old adults from the same dish, with 30 individuals of each sex used for each of the two stress treatments, and 15 individuals of each sex used for the control group. Higher sample sizes were used in the stress treatments to compensate for potential mortality. No food was provided during the acute stress treatment, and cultures were kept in a 20 °C incubator. Previous time-series exposure experiments to H_2_O_2_ in *T. japonicus* showed that expression of the oxidative stress responding biomarker glutathione S-transferase Sigma-class (GST-S) was down-regulated within the first hour and then increased until 6 h or later post exposure [60–62]. To capture the early transcriptomic response in *T. californicus*, two time points (3 h and 6 h) after the start of the experiment were chosen. Four individuals of each sex were sampled from each group. Each individual was dried on a piece of filter paper for one to two seconds and then transferred into a 2 mL nuclease-free tubes with 30–50 1.1 mm diameter zirconia/silica beads (BioSpec Products, USA). The egg sacs of gravid females were removed on the filter paper using dissecting needles and excluded from further sampling. Samples were flash-frozen in liquid nitrogen and stored at − 80 °C until RNA extraction. No individuals died during the exposure experiments.

### Transcriptome sequencing

RNA-seq was performed on individual animals to avoid pooling bias (e.g. [47]) and to assess expression variance among individuals. RNA extraction was started with the addition of 300 μL TRIzol Reagent (Ambion, USA) and homogenization by TissueLyser (Qiagen, USA). The Direct-zol RNA MicroPrep Plus kit (Zymo Research, USA) was utilized for further extraction steps including in-column DNase I treatment, and finally 15 μL nuclease-free water was added to elute RNA from the column. RNA was stored at − 80 °C until preparation of RNA sequencing libraries. Forty-eight libraries were constructed according to the LM-Seq protocol [67]. For details of single-individual RNA-seq library preparation, please refer to our previous study [35]. One modification was made in the protocol to use 6-digit index primers (Illumina RNA PCR Index Primers RPI1-RPI24) to replace the 10-digit index primers due to the small number of libraries in each sequencing lane and the common settings for demultiplexing libraries. As in our previous work [35], 20 cycles of PCR were used for library preparation, since preliminary assays with fewer cycles did not generate sufficient products for construction of sequencing libraries. Additional dual size-selection was conducted by AMPure XP beads (Beckman Coulter, USA). Final libraries were quantified using a Qubit dsDNA HS Assay Kit (Invitrogen, USA) and quality-assessed by Bioanalyzer 2100 system (Agilent, USA). Twenty-four libraries from each time point were pooled together based on their molar concentration for sequencing in one lane. All 48 libraries were sequenced by 150 bp paired-end reads in two lanes of the Illumina HiSeq 4000 platform at Fulgent Genetics (CA, USA).

### Differential expression analysis

Adapter removal, quality trimming and length trimming were performed using Trimmomatic v0.38 with default parameters [68]. After evaluation by FastQC v0.11.8, trimmed reads were mapped to the San Diego population reference genome with the accession number of GCA_007210705.1 [22] by HISAT2 v2.1.0 [69], and StringTie v1.3.4d [70] was used to estimate the count value for each annotated gene. PCA was conducted to identify a possible outlier if its PC eigenvalue was more than three standard deviations away from the mean value for at least one of the first five PCs. DESeq2 [71] was utilized for differential expression analysis between sexes as well as between oxidative stress treated groups and control groups at each time point within each sex. *P*-values were adjusted by the Benjamini and Hochberg (BH) procedure [72] to control the false discovery rate. Genes with adjusted *P*-value < 0.05 and |fold change| > 2 were considered as differentially expressed genes (DEGs). Gene ontology (GO) enrichment analyses of differentially up-regulated and down-regulated genes was conducted by topGO [73] in R.

### Partitioning of gene expression variation

Genes with half of the individuals having less than 1 read per million mapped reads were filtered out, and then normalized expression values for the remaining genes were log2 transformed. A total of 6511 and 6098 genes were kept for the 3 h and 6 h time points, respectively. Variance in gene expression was partitioned by the R package variancePartition version 1.12.1 [74] to estimate the variance explained by effects including sex, treatment and individual replicate at each time point. Detailed methods were described in our previous study [35]. Briefly, the nested analysis of variance (ANOVA) model for each gene was assumed as:

$$ {\mathrm{Y}}_{\mathrm{i}\mathrm{j}\mathrm{k}}=\upmu +{\mathrm{T}}_{\mathrm{i}}+{\mathrm{S}}_{\mathrm{j}\left(\mathrm{i}\right)}+{\mathrm{R}}_{\mathrm{k}\left(\mathrm{i}\mathrm{j}\right)}+{\mathrm{e}}_{\mathrm{i}\mathrm{j}\mathrm{k}} $$

where μ represents the mean expression level for the given gene, and Y_ijk_ is the normalized expression level in the k^th^ replicate (R) which is nested within j^th^ sex (S), which is nested within i^th^ treatment (T). The proportion of variance explained by each variable was measured as the ratio of the variance due to the corresponding variable to the total variance due to all three variables: σ^2^ / (σ_T_^2^ + σ_S_^2^ + σ_R_^2^). An average percentage of variance due to each effect was calculated for each time point.

## Supplementary Information

**Additional file 1.**

## Data Availability

The sequences generated during this study were deposited at the National Center for Biotechnology Information (NCBI) Sequence Read Archive (SRA) under the accession numbers SRR10444974 – SRR10445021. The reference genome used in our analyses is available with the GenBank assembly accession of GCA_007210705.1, and the genome assembly version is Tcal_SD_v2.1.

## Abbreviations

ROS: Reactive Oxygen Species
GST: Glutathione S-Transferase
LM-seq: Ligation Mediated RNA sequencing
PCA: Principal Component Analysis
DEG: Differentially Expressed Gene
GO: Gene Ontology
